# Automatic measurement of short-term variability of repolarization to indicate ventricular arrhythmias in a porcine model of cardiac ischaemia

**DOI:** 10.1093/europace/euad341

**Published:** 2023-11-09

**Authors:** Vera Loen, Agnieszka Smoczynska, Alfonso Aranda Hernandez, Coert O S Scheerder, Britt H R van der Linde, Henriëtte D M Beekman, Aina Cervera-Barea, Gerard J J Boink, Joost P G Sluijter, Marcel A G van der Heyden, Mathias Meine, Marc A Vos

**Affiliations:** Department of Medical Physiology, University Medical Center Utrecht, Yalelaan 50, 3584 CM Utrecht, The Netherlands; Department of Cardiology, University Medical Center Utrecht, Utrecht, The Netherlands; Cardiac Rhythm Management, Medtronic Inc., Mounds View, MN, USA; CRM EMEA Medical Science, Medtronic Bakken Research Center, Maastricht, The Netherlands; Department of Medical Physiology, University Medical Center Utrecht, Yalelaan 50, 3584 CM Utrecht, The Netherlands; Department of Medical Physiology, University Medical Center Utrecht, Yalelaan 50, 3584 CM Utrecht, The Netherlands; Experimental Cardiology Laboratory, Department of Cardiology, University Medical Center Utrecht, Utrecht, The Netherlands; Department of Medical Biology, Amsterdam Cardiovascular Sciences, Amsterdam University Medical Center, Amsterdam, The Netherlands; Department of Medical Biology, Amsterdam Cardiovascular Sciences, Amsterdam University Medical Center, Amsterdam, The Netherlands; Department of Cardiology, Amsterdam Cardiovascular Sciences, Amsterdam University Medical Center, Amsterdam, The Netherlands; Experimental Cardiology Laboratory, Department of Cardiology, University Medical Center Utrecht, Utrecht, The Netherlands; Department of Medical Physiology, University Medical Center Utrecht, Yalelaan 50, 3584 CM Utrecht, The Netherlands; Department of Cardiology, University Medical Center Utrecht, Utrecht, The Netherlands; Department of Medical Physiology, University Medical Center Utrecht, Yalelaan 50, 3584 CM Utrecht, The Netherlands

**Keywords:** Short-term variability of repolarization, Risk monitoring, Ventricular arrhythmias, Cardiac ischaemia, Intracardiac electrogram, Automatic measurement, Translational research

## Abstract

**Aims:**

An automated method for determination of short-term variability (STV) of repolarization on intracardiac electrograms (STV-ARI_auto_) has previously been developed for arrhythmic risk monitoring by cardiac implantable devices, and has proved effective in predicting ventricular arrhythmias (VA) and guiding preventive high-rate pacing (HRP) in a canine model. Current study aimed to assess (i) STV-ARI_auto_ in relation to VA occurrence and secondarily (ii-a) to confirm the predictive capacity of STV from the QT interval and (ii-b) explore the effect of HRP on arrhythmic outcomes in a porcine model of acute myocardial infarction (MI).

**Methods and results:**

Myocardial infarction was induced in 15 pigs. In 7/15 pigs, STV-QT was assessed at baseline, occlusion, 1 min before VA, and just before VA. Eight of the 15 pigs were additionally monitored with an electrogram catheter in the right ventricle, underwent echocardiography at baseline and reperfusion, and were randomized to paced or control group. Paced group received atrial pacing at 20 beats per min faster than sinus rhythm 1 min after occlusion. Short-term variability increased prior to VA in both STV modalities. The percentage change in STV from baseline to successive timepoints correlated well between STV-QT and STV-ARI_auto_. High-rate pacing did not improve arrhythmic outcomes and was accompanied by a stronger decrease in ejection fraction.

**Conclusion:**

STV-ARI_auto_ values increase before VA onset, alike STV-QT in a porcine model of MI, indicating imminent arrhythmias. This highlights the potential of automatic monitoring of arrhythmic risk by cardiac devices through STV-ARI_auto_ and subsequently initiates preventive strategies. Continuous HRP during onset of acute MI did not improve arrhythmic outcomes.

## Graphical Abstract

**Graphical Abstract:**
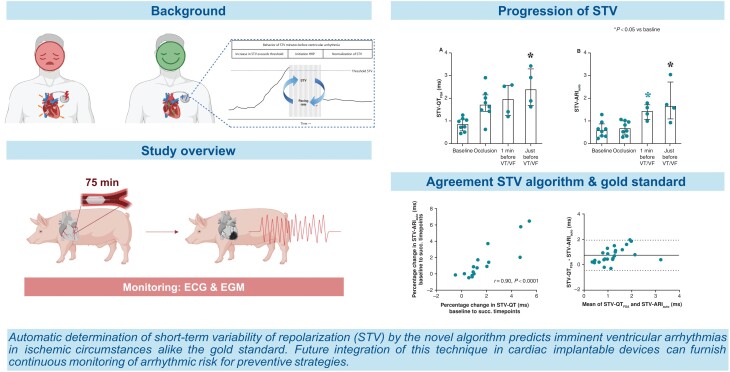

**What’s new?**

- The novel automatic method for measurement of short-term variability (STV) of repolarization on intracardiac electrograms (STV-ARI_auto_) was tested in a porcine model of ischaemia-induced ventricular arrhythmias (VA).
- STV-ARI_auto_ values increase prior to VA, alike the gold-standard STV method.
- Integration of the STV-ARI_auto_ algorithm in cardiac implantable electronic devices could allow for continuous monitoring of arrhythmic risk. Subsequently, preventive strategies may be employed to maintain patient quality of life.

## Introduction

Management of sudden cardiac death (SCD) arising from unexpected ventricular arrhythmias (VA) has swiftly evolved in the last century. The introduction of the implantable cardioverter defibrillator (ICD) notably curbed mortality among high-risk patients, becoming the cornerstone of SCD treatment.^1–3^ Nevertheless, SCD remains a major healthcare concern, necessitating comprehensive research into its underlying mechanisms.^4^ Furthermore, the high prevalence of conditions like depression, anxiety, and post-traumatic stress disorder among ICD patients, especially those undergoing therapy, underscores the importance of developing new approaches and optimizing existing treatments.^5^

The parameter short-term variability (STV) of repolarization, which quantifies beat-to-beat repolarization differences, has been proposed as a marker for arrhythmic risk monitoring, both short- and long-terms. In the chronic atrioventricular block (CAVB) dog model, in which Torsade de Pointes (TdP) arrhythmias can be provoked by pharmacological block of repolarizing currents following volume-overload-related cardiac remodelling,^6^ STV at baseline identified subjects that developed SCD during follow-up.^7^ Moreover, STV increased shortly before drug-induced TdP, predicting imminent VA.^7,8^ Similar results were obtained in humans, where elevated STV at baseline was associated with the occurrence of VA in patients with long QT syndrome, non-ischaemic cardiomyopathy, and structural heart disease, and could predict impending VA.^9–14^ To continuously monitor the arrhythmic risk by cardiac implantable devices, a fully automatic method to determine STV on the intracardiac electrogram (STV-ARI_auto_) has been developed. This method demonstrated high efficacy in predicting VA in the CAVB dog model.^15^ Subsequently a proof-of-principle study was performed, where high-rate pacing (HRP) was initiated by the ICD, guided by the automatically measured STV values. The technique was very effective, preventing 7/8 episodes.^16^ To which extent the STV-ARI_auto_ application is feasible in conditions beyond the CAVB dog, in which VA are mostly initiated by focal activity, needs to be established.^17^ Of significant note is that the majority of ICD implants is in a background of ischaemic cardiomyopathy.^18,19^

Recently, the suitability of STV derived from the ECG (STV-QT) to forecast impending VA in a porcine model of myocardial infarction (MI) was shown.^20^ This suggests that devices could be employed for risk assessment through STV monitoring in ischaemic circumstances as well, if STV is measured automatically and reliably. In acute MI, current views are that arrhythmia induction during the initial phases Ia and Ib is characterized by early and delayed after depolarization-mediated ectopy and functional re-entry.^21,22^ The first mechanism is likely to respond to rapid pacing.^23^ On the other hand, pacing aggravates myocardial ischaemia and infarct size associates with vulnerability for VA development.^20,24^

As arrhythmia and SCD risk stratification remain challenging after MI,^4^ the primary aim of this proof-of-principle study was to assess the performance of the novel automatic method of arrhythmic risk monitoring through STV-ARI_auto_ measurements in a porcine model of ischaemia-induced VA. Secondary aims were to confirm the observation that STV-QT can predict impending VA,^20^ establishing STV-QT as the gold-standard for the primary study aim, and to preliminarily explore the effect of HRP on the occurrence of arrhythmias in ischaemic circumstances.

## Methods

### Animals

All experiments were performed in accordance with the ‘Directive 2010/63/EU of the European Parliament and of the Council of 22 September 2010 on the protection of animals used for scientific purposes’ and the Dutch law, laid down in the Experiments on Animals Act (license AVD1150020172624 and AVD1180020198304). Myocardial infarction was experimentally induced in 15 adult pigs with a median weight of 63.5 ± 23 kg (Topigs Norsvin, Lelystad, The Netherlands). Two experimental sets of seven pigs (Part 1, four females and three males) and eight pigs (Part 2, all females) respectively were performed (*Table 1*).

**Table 1:** Animal characteristics of Part 1 and Part 2

	Part 1	Part 2		
	All subjects	All subjects	Control	Paced
Pigs (*n*)	7	8	4	4
Female sex	4	8	4	4
Weight (kg)	61.5 ± 23.0	65.0 ± 10.5	65.0 ± 3.5	63.5 ± 10.5
Time to first VT/VF (min)	23.0 ± 54.0	15.6 ± 72.1	17.2 ± 64.5	10.1 ± 11.6
Defibrillation shocks (*n*)	3 ± 11	10 ± 41	8 ± 12	14 ± 35

Data expressed as median ± interquartile range or *n*. No statistical differences were found between Part 1 and Part 2, nor between control and paced subjects of Part 2.

kg, kilograms; VT, ventricular tachycardia; VF, ventricular fibrillation; min, minutes.

**Figure 1:**
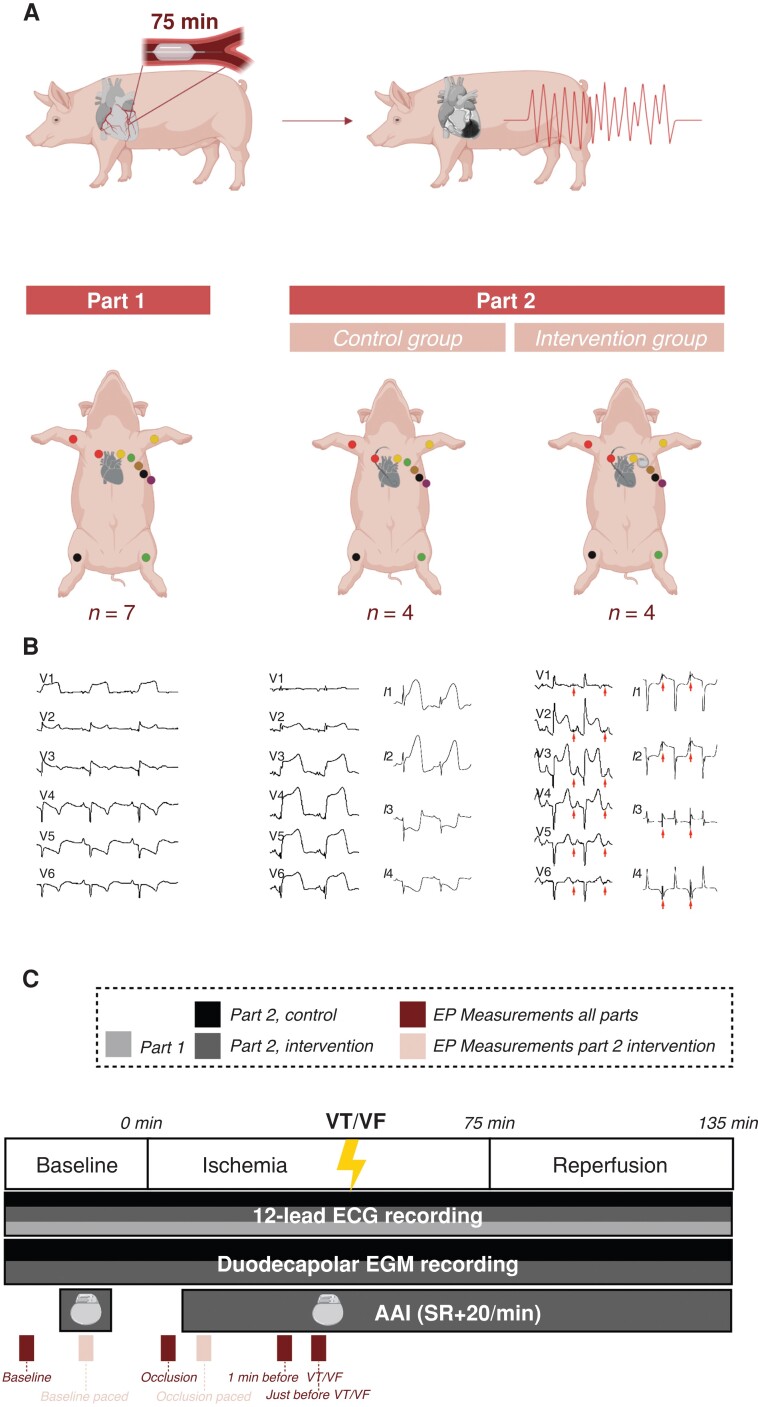
Overview of experimental setup. (*A*, top) Schematic overview of occlusion of the mid LAD coronary artery to induce VT/VF (*A*, bottom) illustration of electrode placement for the 12-lead ECG for all study parts, addition of an EGM catheter in Part 2 and a pacemaker for the intervention group of Part 2. (*B*) Representative traced of the precordial leads of the ECG and first four EGM vectors (EGM in Part 2 only) during ischaemia. Arrows indicate pacing spikes. (*C*) Experimental design and timepoint taken for assessment of EP parameters. LAD, left anterior descending coronary artery; VT, ventricular tachycardia; VF, ventricular fibrillation; EP, electrophysiological; AAI [SR + 20/min], atrial demand pacing at 20 beats per min faster than sinus rhythm.

Part 1 was part of an ongoing study and involved assessment of the progression of STV-QT calculated with the technique of fiducial segment averaging (STV-QT_FSA_),^25^ starting from occlusion until the occurrence of the first VA episode. Part 2, in addition to STV-QT_FSA_, assessed STV-ARI_auto_. Moreover, pigs of Part 2 were randomized in a control (*n* = 4) and intervention group (*n* = 4) to assess the influence of HRP on arrhythmic outcomes (*Figure 1*).

### Surgical preparation

Starting 10 days before the experiment, female pigs were given daily doses of amiodarone (first dose 1200 mg, then 800 mg) and clopidogrel (75 mg). Males (3/7 pigs of Part 1) received daily doses of dronedarone (400 mg) starting 2 days before the experiment. One day before the infarction, all pigs received a transdermal buprenorphine patch (35 µg/h) and females received acetylsalicylic acid (first dose 320 mg, then 80 mg). On the day of the experiment, pigs were pre-medicated with ketamine [intramuscular (im); 10 mg/kg], midazolam (im; 0.5 mg/kg), and atropine (im; 0.05 mg/kg). General anaesthesia was induced with intravenous thiopental (4 mg/kg) and maintained by continuous intravenous (iv) infusion of cisatracurium (0.7 mg/kg/h), midazolam (0.4 mg/kg/h), and sufentanil (2.5 µg/kg/h). All animals received amiodarone (300 mg) during 45 min after induction of general anaesthesia.

Pigs were mechanically ventilated at a respiratory rate of 12/min and a tidal volume of approximately 10 ml/kg, adjusted to maintain end-tidal CO_2_ pressures between 35 and 45 mmHg, using a mixture of oxygen and air (FiO_2_ 0.5). Heparin (5000 IE) was administered prior to cannulation of the carotid artery, balloon occlusion, and reperfusion. Blood pressure was continuously monitored by an arterial pressure catheter in the left or right femoral artery, depending on vessel patency.

### Induction of myocardial infarction

With the pig in a supine position, venous and arterial 8-French sheaths were placed in the carotid artery and jugular vein. Subsequently, coronary angiography (CAG) was conducted to delineate the anatomy of the left anterior descending coronary artery (LAD). A 3.0 mm balloon catheter (Sapphire II Pro, OrbusNeich, Hong Kong) was positioned either after the first or second diagonal branch, taking into consideration the coronary artery anatomy to induce sufficient ischaemia while minimizing the risk of death caused by refractory ventricular fibrillation (VF) during the experiment. The balloon catheter was pressurized to 6 bar to induce occlusion, and successful occlusion was verified through CAG. In females, following 75 min of occlusion, the catheter was removed, allowing for a 60 min reperfusion period (*Figure 1*). Males sustained a pre-conditioning period of three times 5 min occlusion, followed by a permanent occlusion of 120 min until reperfusion.

Haemodynamically intolerated VA, i.e. VF and sustained ventricular tachycardia (VT), were terminated by applying 200 J shocks using the external paddles of a Heartstart XL defibrillator (Phillips, The Netherlands). During every episode, a dose of amiodarone (150 mg) was administered with a maximum of three consecutive administrations.

### Electrophysiology and electrocardiography

#### Part 1—behaviour of STV-QT_FSA_

After inducing anaesthesia, a 12-lead ECG (Cardioperfect, Welch Allyn, USA) at a sampling frequency of 1200 Hz and filtering to a bandwidth of 0.05–1200 Hz was recorded continuously until termination of the experiment.

#### Part 2—assessment of STV-ARI_auto_ and high-rate pacing

In addition to the ECG recordings, echocardiography was performed under anaesthesia (Philips iE33 ultrasound, Best, The Netherlands) at baseline and after reperfusion. Animals were positioned in right-sided supine position and apical four-chamber views were obtained, while porcine anatomy did not allow for apical two-chamber views. Moreover, a duodecapolar electrogram (EGM) catheter (St. Jude Medical, St. Paul, MN, USA) and a temporary pacing lead (Hugo Sachs Elektronik, March, Germany) were respectively placed in the right ventricle and right atrium under fluoroscopic guidance. Electrogram catheters were configured unipolarly with the reference electrode in the femoral vein and were recorded with EP Tracer (Cardiotek, Maastricht, The Netherlands) at a sampling frequency of 1000 Hz. The four pigs that were assigned to the intervention group were paced for 5 min at baseline and from 1 min after occlusion until the end of the experiment (*Figure 1*). The pacing frequency was set to the heart rate measured at baseline, increased by 20 beats per min. The four pigs of the control group were not paced during the experiment.

### Data analysis

#### Part 1—behaviour of STV-QT_FSA_

**Figure 2:**
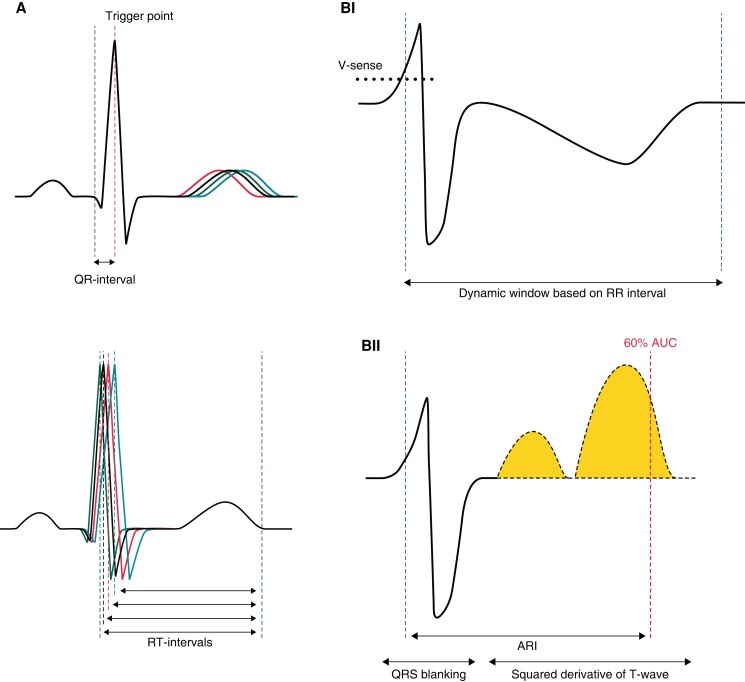
Schematic overview of techniques to measure short-term variability (STV) of repolarization. (*A*) STV-QT_FSA_ measured with fiducial segment averaging (FSA) on the ECG. Complexes were aligned at the R peak, onset of the QRS complex, and the T-wave end to determine QT interval for each individual beat. (*BI*) STV-ARI_auto_ measured with the new automatic method on the EGM. Ventricular sensing indicates the start of repolarization and establishes a dynamic window based on the RR interval. (*BII*) After blanking of the QRS, ARI offset is determined as 60% of the area under the curve (AUC) of the first derivative of the T-wave.

The number of defibrillation shocks and time to the first haemodynamically intolerated VT/VF were registered. RR, QT, and ST-deviation were measured offline with calipers in lead V2. QT interval was corrected for heart rate (QTc) with the Bazett formula. STV-QT_FSA_ was calculated by fiducial segment averaging (FSA).^25^ Briefly, each QRS complex was aligned around the R peak by cross-correlating the individual complex with the average of the other complexes, shifting until maximal correlation was achieved. The same alignment process was repeated for the QRS-onset and the end of the T-wave. Correct alignment was checked visually and manually adjusted where necessary. Subsequently, the RR and QT intervals could be calculated between the fiducial points for each individual beat (*Figure 2A*). Lead V2 was used, unless a very flat T-wave was present and V1 or V3 was selected based on the best trade-off between sufficient T-wave amplitude and limited ST-deviation. Subsequently, STV-QT_FSA_ of 31 consecutive beats was calculated using the formula STV = ∑ [*D n* + 1 − *D n*]/(30 × √2), where *D* represents the determinant of repolarization, i.e. the QT interval and *n* the number of beats taken into account.^26^ Ectopic beats, the beat before, and the two beats after the ectopic beat were manually excluded from analysis. Measurements were performed at baseline, 30 s after occlusion, 1 min before the first VT/VF, and just prior to the first VT/VF episode on ECG segments that were free of noise, displayed a stable morphology, and little ectopy (*Figure 1*).

#### Part 2—assessment of STV-ARI_auto_ and high-rate pacing

In addition to RR, QT(c), ST-deviation, and STV-QT_FSA_, STV-ARI_auto_ values were calculated at the same timepoints on the most apical pole of the duodecapolar EGM that was free of significant ST-deviations and displayed sufficient T-wave amplitude (≥0.5 mV), usually I2 or I3. I1 could not be used due to major injury current. As described previously,^15^ automatic measurement of the activation recovery interval (ARI) started by identification of a complex using device ventricular sensing. In addition to previous work, a dynamic window was created for each beat based on its RR interval (*Figure 2BI*). This window allowed the entire T-wave to be considered without being affected by the adjacent beats, while adapting to different heart rates. The QRS complex was blanked to avoid interference in the T-wave end detection and was set to 200 ms (*Figure 2BII*). After blanking of the QRS, the first derivative of the resulting signal was computed over time to detect changes in the slope. This gradient signal was squared to make all data points positive and to emphasize changes in the signal. The ARI was defined as the time difference between the point representing 60% of the area under the curve of the resultant signal and ventricular sensing. STV-ARI_auto_ was calculated as described above,^26^ in this case *D* representing the ARI.

In the paced pigs of the intervention group, the timepoints ‘baseline paced’ and ‘occlusion paced’ were added. However, significant interference of the pace spike in the T-wave end rendered calculation of QT, QTc, STV-QT_FSA_, and STV-ARI_auto_ impossible on paced signals (*Figure 1*). Therefore, these parameters were only assessed on unpaced signals, i.e. baseline SR and occlusion SR in the intervention group. RR interval and ST-deviation were calculated at baseline, baseline paced, occlusion, occlusion paced, 1 min before VT/VF, and just before VT/VF (*Table 3*, *Figure 4*). Again, ECG and EGM segments at these timepoints were selected that were free of noise, displayed a stable morphology, and little ectopy.

Of the available data, the percentage change in STV-ARI_auto_ from baseline to occlusion, 1 min before VT/VF, and just prior to VT/VF was compared to the percentage changes in STV-QT_FSA_. Time to first VT/VF, number of shocks, and available electrophysiological parameters were compared between control and paced groups.

Ejection fraction (EF) was assessed on four-chamber recordings with the single plane method of disks technique (MOD-sp4), based on left ventricular (LV) end diastolic dimensions vs. LV end systolic dimensions (IntelliSpace Cardiovascular, Philips, Best, The Netherlands) of three consecutive beats. As apical two-chamber views could not be obtained, the Simpson biplane method could not be applied.^27^ The percentage decline in EF from baseline to reperfusion was compared between control and paced groups.

### Statistics

Numerical values are displayed as median ± interquartile range or mean ± standard deviation when passing normality tests. Comparison between groups was done with a two-tailed Mann–Whitney *U* test. For comparison of EF between control and intervention groups, a one-tailed test was used, as evidence is present about the aggravating effect of elevated heart rates on ischaemia.^24^ Within-group comparisons were done with a paired one-way analysis of variance with Dunnett’s correction for multiple comparisons or Kruskwal–Wallis test, depending on normality of the data. The association between STV-ARI_auto_ and STV-QT_FSA_ values was assessed by Spearman correlation. Bland–Altman analysis was done to evaluate systematic bias and limits of agreements. Statistical analyses were performed with GraphPad Prism 8.0 (GraphPad Software Inc., La Jolla, CA, USA). Results were considered statistically significant if *P* < 0.05.

## Results

All animals underwent the protocolized occlusion and reperfusion periods. Angiography showed complete occlusion of the LAD after inflation and return of the blood flow after deflation of the balloon catheter.

### Part 1—behaviour of STV-QT

#### Arrhythmia characteristics

During the occlusion period, pigs received 3 ± 11 defibrillation shocks and median time to the first haemodynamically intolerated VA was 23.0 ± 54.0 min (*Table 1*).

#### Electrophysiology

Electrophysiological parameters measured at the specified timepoints are summarized in *Table 2*. RR intervals and ST-deviation did not change significantly over the course of these experiments. QT interval prolonged from baseline to occlusion (448 ± 69 ms vs. 499 ± 52 ms, *P* < 0.05) and shortened just before VT/VF (431 ± 66 ms, *P* < 0.05 compared to baseline and occlusion). After correcting the QT interval for heart rate, the prolongation from baseline to occlusion is preserved (473 ± 23 ms vs. 532 ± 41 ms, *P* < 0.05), while shortening just before VT/VF only remained significant compared to occlusion (457 ± 50 ms, *P* < 0.05).

**Table 2:** Electrophysiological parameters of Part 1

*n* = 7	Baseline	Occlusion	1 min before to VT/VF	Just before VT/VF
RR (ms)	901 ± 216	888 ± 144	888 ± 142	889 ± 116
QT (ms)	448 ± 69	499 ± 52*	473 ± 71	431 ± 66^*,‡^
QTc (ms)	473 ± 23	532 ± 41*	503 ± 49	457 ± 50^‡^
ST-deviation (mV)	0.02 ± 0.02	0.06 ± 0.07	0.12 ± 0.10	0.13 ± 0.11
STV-QT_FSA_ (ms)	0.75 ± 0.23	1.06 ± 0.25*	2.03 ± 0.61^*,‡^	2.61 ± 1.29^*,‡^

Data expressed as mean ± SD.

VT, ventricular tachycardia; VF, ventricular fibrillation; STV-QT_FSA_, short-term variability derived from the QT interval using the method of fiducial segment averaging.

*P < 0.05 compared to baseline.

^‡^ *P* < 0.05 compared to occlusion.

#### Progression of STV-QT_FSA_

**Figure 3:**
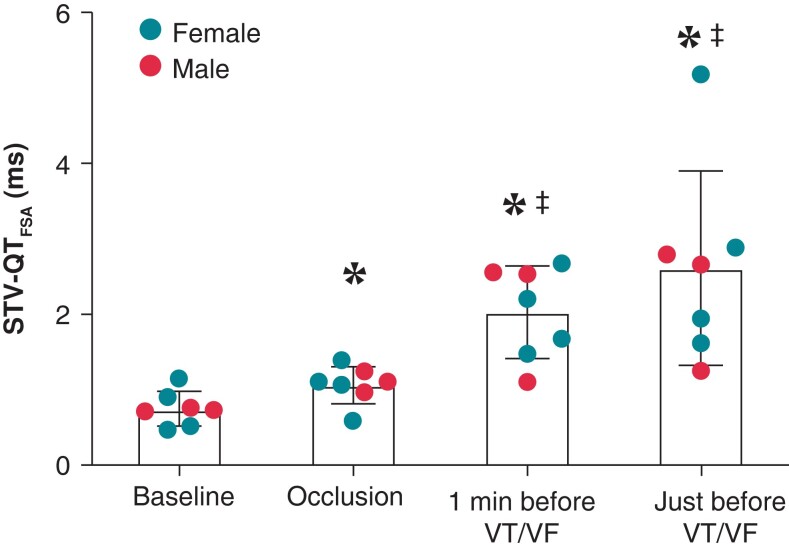
Temporal changes in STV during ischaemia of Part 1. STV-QT_FSA_ increases before VF or VT during ischaemia. Data expressed as mean (bar) ± SD (brackets). *P < 0.05 vs. baseline; ‡<0.05 vs. occlusion. STV-QT_FSA_, short-term variability of the QT interval calculated with fiducial segment averaging; VF, ventricular fibrillation; VT, ventricular tachycardia.

STV-QT_FSA_ increased from baseline compared to occlusion, 1 min before VT/VF, and just before VT/VF (0.75 ± 0.23 vs. 1.06 ± 0.25, 2.03 ± 0.61, and 2.61 ± 1.29 ms, all *P* < 0.05). The increase in STV-QT_FSA_ from occlusion vs. 1 min before and just before VT/VF reached significance as well (*Table 2*, *Figure 3*).

### Part 2—assessment of STV-ARI_auto_ and high-rate pacing

#### Arrhythmia characteristics

Ventricular fibrillation occurred in seven of eight pigs, while one subject from the control group displayed a VT with haemodynamic instability during reperfusion. Each subject received a median of 10 ± 41 defibrillation shocks, and time to the first haemodynamic unstable episode was 15.6 ± 72.1 min (*Table 1*).

#### Electrophysiology

In *Table 3*, the electrophysiological parameters measured at the specified timepoints are shown for all pigs of Part 2 and split up in control and intervention groups, if measured due to the above-mentioned interference of the pace spike in the T-wave. In contrast to Part 1, no differences in QT or QTc were found and ST-deviation on ECG and EGM increased from baseline to 1 min before VT/VF and just before VT/VF.

**Table 3:** Electrophysiological parameters of Part 2

All subjects (*n* = 8)^a^		Baseline (SR)	Baseline (paced)	Occlusion (SR)	Occlusion (paced)	1 min before to VT/VF	Just before VT/VF
	RR (ms)	725 ± 249		736 ± 335		642 ± 226	637 ± 258
	QT (ms)	432 ± 126		470 ± 119		*439 ± 94*	*438 ± 112*
	QTc (ms)	512 ± 69		507 ± 115		*518 ± 62*	*516 ± 67*
	ST-dev ECG (mV)	0.02 ± 0.05		0.08 ± 0.26		0.16 ± 0.19*	0.13 ± 0.27*
	ST-dev EGM (mV)	0.02 ± 0.36		0.15 ± 0.52		0.28 ± 0.55*	0.33 ± 0.62*
	STV-QT_FSA_ (ms)	0.87 ± 0.81		1.72 ± 2.28		*1.97 ± 1.39*	2.40 ± 1.79*
	STV-ARI_auto_ (ms)	0.58 ± 1.04		0.69 ± 0.78		1.43 ± 0.75*	1.65 ± 2.10*
						1 min prior to VT/VF (SR)	Just before VT/VF (SR)
Control group (*n* = 4)	RR (ms)	711 ± 249		691 ± 325		714 ± 136^#^	719 ± 179^#^
	QT (ms)	441 ± 126		438 ± 90		439 ± 94	438 ± 112
	QTc (ms)	523 ± 60		507 ± 49		518 ± 62	516 ± 67
	ST-dev ECG (mV)	0.04 ± 0.04		0.05 ± 0.26		0.16 ± 0.14	0.13 ± 0.27
	ST-dev EGM (mV)	0.04 ± 0.04		0.26 ± 0.45		0.40 ± 0.55	0.36 ± 0.62
	STV-QT_FSA_ (ms)	0.86 ± 0.65		1.92 ± 0.77		1.97 ± 1.39	2.40 ± 1.79*
	STV-ARI_auto_ (ms)	0.59 ± 1.04		0.69 ± 0.49		1.43 ± 0.75	1.65 ± 2.10
						1 min prior to VT/VF (paced)	Just before VT/VF (paced)
Intervention group (*n* = 4)	RR (ms)	725 ± 60	593 ± 48	764 ± 240	593 ± 48	593 ± 48^#^	593 ± 48^#^
	QT (ms)	432 ± 66	—	471 ± 95	—	—	—
	QTc (ms)	501 ± 69	—	523 ± 115	—	—	—
	ST-dev ECG (mV)	0.02 ± 0.04	0.01 ± 0.03	0.08 ± 0.10	0.14 ± 0.14	0.21 ± 0.19	0.17 ± 0.17
	ST-dev EGM (mV)	0.02 ± 0.10	0.12 ± 0.24	0.15 ± 0.21	0.12 ± 0.06	0.28 ± 0.14*	0.33 ± 0.32*
	STV-QT_FSA_ (ms)	0.90 ± 0.75	—	1.56 ± 2.28	—	—	—
	STV-ARI_auto_ (ms)	0.49 ± 0.62	—	0.64 ± 0.78	—	—	—

Data expressed as median ± interquartile range.

ST-dev, ST-deviation; SR, sinus rhythm; VT, ventricular tachycardia; VF, ventricular fibrillation; STV-QT_FSA_, short-term variability derived from the QT interval using the method of fiducial segment averaging; ARI, activation recovery interval; STV-ARI_auto_, short-term variability derived from the activation recovery interval using the new automatic method.

*P < 0.05 compared to baseline (SR).

^#^ *P* < 0.05 intervention group compared to control group same measurement.

^a^Data in italic font represent measurements from the control group only (*n* = 4) due to pace spike interference in the T-wave.

**Figure 4:**
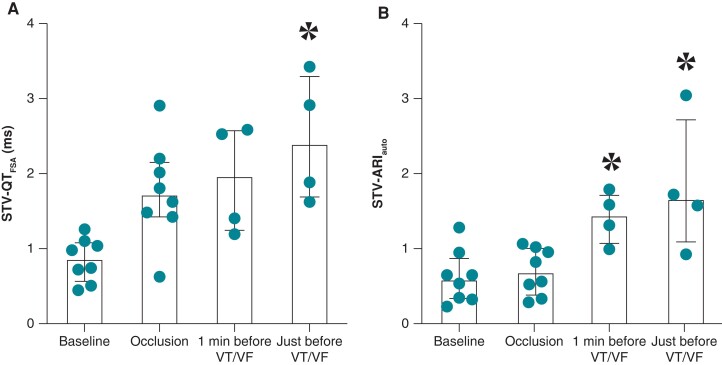
Temporal changes in STV during ischaemia of Part 2. (*A*) STV-QT_FSA_ increases before VF or VT during ischaemia. (*B*) STV-ARI_auto_ increases prior to VF or VT during ischaemia. Data expressed as median (bar) ± interquartile range (brackets). Data 1 min before VT/VF and just before VT/VF represent measurements from the control group only (*n* = 4) due to pace spike interference in the T-wave. *P < 0.05 vs. baseline; STV-QT_FSA_, short-term variability (STV) of the QT interval calculated with fiducial segment averaging; STV-ARI_auto_, STV of the activation recovery interval (ARI) of intracardiac electrograms calculated with the new automatic method; VF, ventricular fibrillation; VT, ventricular tachycardia.

Both STV-QT_FSA_ and STV-ARI_auto_ increased from baseline (0.87 ± 0.81 and 0.58 ± 1.04 ms, *n* = 8) to just before VT/VF (2.40 ± 1.79 and 1.65 ± 2.10 ms, both *P* < 0.05 and *n* = 4). Additionally, STV-ARI_auto_ increased from baseline to 1 min before VT/VF as well (*Figure 4*).

#### Agreement between STV-ARI_auto_ and STV-QT_FSA_

**Figure 5:**
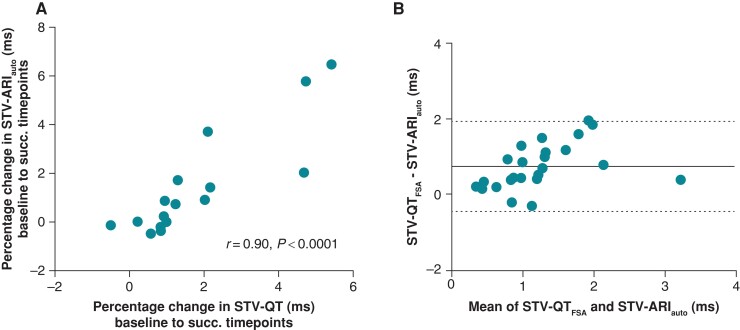
Comparison of STV-QT_FSA_ and STV-ARI_auto_ values. (*A*) Spearman’s correlation coefficient of the percentage changes from baseline to the successive three timepoints between STV-QT_FSA_ and STV-ARI_EGM_. (*B*) Bland–Altman analysis comparing the difference between STV-QT_FSA_ and STV-ARI_auto_ to the mean. STV-QT_FSA_, short-term variability (STV) of the QT interval calculated with fiducial segment averaging; STV-ARI_auto_, STV of the activation recovery interval (ARI) of intracardiac electrograms calculated with the new automatic method; succ., successive.

The percentage change from baseline to occlusion, 1 min before VT/VF, and just before VT/VF showed a high association between STV-ARI_auto_ and STV-QT_FSA_ (Spearman’s *r* = 0.90, *P* < 0.0001, *Figure 5A*). A systematic bias was found of 0.75 ms, and the limits of agreement of the Bland–Altman plot were −0.43 and 1.93 ms (*Figure 5B*).

#### Effect of high-rate pacing

**Figure 6:**
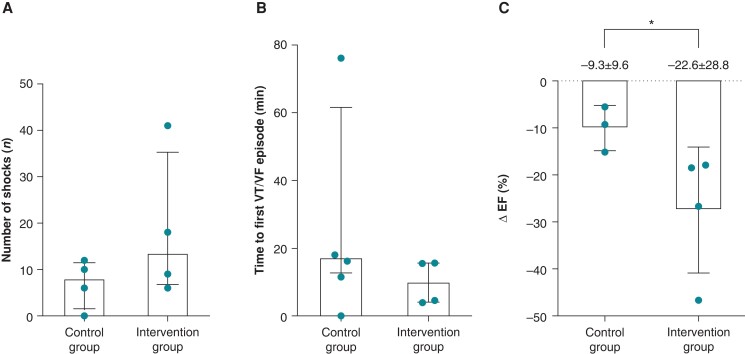
Comparison between control and intervention groups. (*A*) Number of defibrillation shocks administered. (*B*) Median time to developing the first VT or VF episode. (*C*) Percentual change in EF measured at baseline vs. after 1 h of reperfusion. Data expressed as median (bar) ± interquartile range (brackets). *P < 0.05. EF, ejection fraction; VT, ventricular tachycardia; VF, ventricular fibrillation.

The time between occlusion and the first VT/VF was 17.2 ± 64.5 vs. 10.1 ± 11.6 min for the control and intervention groups, respectively (*Figure 6A*). The total number of shocks administered to the four pigs in the control group was 28 with a median of 8 ± 12 shocks per pig. In contrast, the total number of shocks administered in the intervention group was 74 shocks with a mean of 14 ± 35 shocks per pig (*Figure 6B*). The percentage change in EF (ΔEF) from baseline to reperfusion was −9.3 ± 9.6% vs. −22.6 ± 28.8% in the control vs. intervention group, respectively (*P* < 0.05, *Figure 6C*). One subject from the control group was omitted from this analysis due to technical difficulties.

## Discussion

The current work assessed the evolution of STV, calculated with the novel automatic algorithm and the gold-standard derived from the QT interval, in relation to the occurrence of VA and the effect of HRP on arrhythmic outcomes in a porcine model of acute ischaemia/reperfusion. The primary finding was that automatic monitoring of the arrhythmic potential through STV-ARI_auto_ measurements can indicate impending VA during acute MI. Secondary outcomes were confirmation that STV-QT_FSA_ increased abruptly prior to VA as well and that rapid pacing during phase Ia of MI did not improve arrhythmic outcomes, which was accompanied by a more pronounced decrease in EF.

### Short-term variability for arrhythmic potential monitoring

Extensive previous work has established that STV outperforms parameters based solely on repolarization duration to assess arrhythmic risk. In the CAVB dog model, an increase in STV before VA occurrence is consistently documented and linked to arrhythmia severity, in contrast to repolarization duration.^6,8^ These patterns hold true in human studies as well: in patients with ischaemic and non-ischaemic cardiomyopathy, STV-QT increased just before VA, while QT and QTc-intervals did not.^12,13^ Therefore, the findings in the current study—indicating that STV, unlike QT(c)-intervals and ST-elevation, signals imminent VA during acute MI—are consistent with literature.

As an association between STV and ST-deviation was demonstrated previously,^20^ the additional value of STV to ST-deviation remained to be elucidated.^20^ In addressing this, lead V2 was chosen for assessment of electrophysiological parameters in the present study, since V2 displayed intermediate ST-deviations in the referred study. Study Part 1 demonstrated additional value, as ST-deviation did not increase significantly over the course of the experiment, unlike STV-QT_FSA_. Part 2 did show significant ST-deviations, presumably because HRP exacerbated cardiac ischaemia.^24^

Besides application of STV-ARI_auto_ measurements for monitoring of imminent arrhythmic risk by ICDs, there are also opportunities for assessment of longer-term arrhythmic risk. In patients with long QT syndrome, non-ischaemic heart failure, and structural heart disease, elevated baseline STV was associated with the occurrence of VA.^9–11,14^ Moreover, circadian STV showed distinct peaks in the morning and evening in ICD patients with elevated arrhythmic events, in contrast to patients with a lower arrhythmic burden.^28^ In a similar vein, variations in ventricular repolarization duration in the low-frequency domain have shown a strong predictive value for VA and SCD in patients with ischaemic and non-ischaemic cardiomyopathy.^29^ As an interaction between low-frequency and beat-to-beat variations in repolarization was found based on alterations in *I*_Kr_, *I*_K1_, and *I*_CaL_ current densities, this emphasizes the potential application of STV measurements to identify patients at risk of experiencing life-threatening VA.^29^ Consequently, the risk of ICD shocks or SCD can be mitigated indirectly by additional diagnostics on the identified high-risk patients and, depending on findings, subsequent relieve of coronary occlusion, substrate ablation, or pharmacological treatment.

In more acute settings like the coronary care unit, automatic STV measurements can be employed to monitor the risk of VA after MI, enabling proactive response from medical staff.

Moreover, employment of automatic STV measurements can aid the selection of eligible ICD candidates, as current guidelines for primary prevention overlook a significant number of patients who will require ICD therapy,^4^ while low annual shock rates on the other hand indicate that many patients may be unnecessarily exposed to the risks and inconveniences of an ICD.^30,31^ As manual STV measurements are time-consuming, automated measurements enhance its feasibility of implementation in screening.

### Different short-term variability methods

The current work demonstrated that STV-ARI_auto_ and STV-QT_FSA_ show similar values and trends, displaying a strong association (*r* = 0.90), a bias of 0.75 ms and acceptable limits of agreement, which underlines the robustness of the automatic STV technique. This is in line with previous work in the CAVB dog, where STV-ARI_auto_ showed a strong association with STV derived from monophasic action potential duration and could accurately predict imminent VA.^15^ The systematically higher STV values derived from the QT interval than the ARI in the current work could be attributed to (i) differences in methodology and (ii) differences in source signal.

Both STV techniques aim to enhance accuracy by minimizing measurement errors for individual beats. The automatic method achieves this by automatically detecting the ARI in a consistent manner for each beat, reducing the impact of human error. By squaring the first derivative of the signal, the changes in T-wave slope receive emphasis, and determination becomes irrespective of T-wave direction (i.e. positive and negative of biphasic). Fiducial segment averaging enables simultaneous determination of fiducial points (R peak, Q onset, and T-wave offset) for all complexes.^25^ The shifts between these fiducial points until maximum correlation is achieved are utilized to calculate individual ARIs. This ensures the preservation of beat-to-beat variations without introducing repetitive measurement errors. However, it is important to note that FSA requires the researcher to identify and visually check the fiducial points, introducing a slight potential for human error.

Since the ECG regards cardiac electrophysiology more globally than the EGM, more intracardiac differences can be reflected in the STV derived from the QT interval, as such producing higher STV values. This is of particular interest for future studies with transvenous ICDs in humans, as there are both near-field and far-field vectors available in the ICD. What’s more, the noise in some ECG recordings could not be filtered out while the EGM recordings were overall more stable and easier to apply additional filtering to. As noise introduces fluctuations in T-wave morphology, it impacts STV. However, the reported STV-QT_FSA_ values are in the same order of magnitude as the STV-QT values derived from lead V2 in earlier work on STV in a porcine MI model.^20^

### High-rate pacing vs. arrhythmic potential

The application of HRP to reduce arrhythmic risk has been well-established in patients with long QT syndrome and pause-dependent VA.^32–34^ This is supported by several studies in the CAVB dog model, where HRP prevented VA in 70–88% and the reduction in arrhythmic potential during pacing was reflected by a decrease in STV.^16,23,35^ The anti-arrhythmic properties of an elevated heart rate lie in amplification of the delayed rectifier potassium current, thus strengthening the repolarization reserve. This effect combined with inactivation of the L-type calcium current inhibits the formation of early after depolarizations, which can trigger an arrhythmia and can facilitate arrhythmia perpetuation through an increased heterogeneity in repolarization.^36,37^

In the current study, starting continuous HRP 1 min after occlusion, i.e. phase Ia of MI, disrupted STV-ARI_auto_ measurements and did not decrease the arrhythmic outcomes. Instead, it showed an inclination towards more and faster occurring VA and reduced ejection fraction after reperfusion, indicating worsened cardiac function due to aggravated ischaemia. Previous clinical and pre-clinical research on the effect of HRP during ischaemia/reperfusion highlights the importance of timing.^24,38–40^ Rapid pacing generally increases myocardial metabolic demand, adapting by increasing oxygen consumption. This is the basis of cardiac pre-conditioning, where hearts paced before ischaemia exhibit smaller infarct sizes.^41^ Conversely, initiating HRP with ischaemia onset exacerbates oxygen deficiency, worsening outcomes.^42^ The current data, despite pacing before occlusion, point to the latter effect. Consequently, STV-guided HRP during acute MI should be avoided. The potential of such pacing to prevent arrhythmias in ischaemic cardiomyopathy, more relevant for ICD patients than acute MI, needs further clarification.

The exact pathophysiological mechanism of spontaneous occurrence of VA in ischaemic cardiomyopathy remains incompletely understood. Following MI, a complex remodelling process takes place. Electrical remodelling leads to a reduced repolarization reserve, abnormal calcium handling, and repolarization inhomogeneity.^43–45^ These factors facilitate both ectopy as triggering event and a functional substrate. Sympathetic hyperresponsiveness, scar formation, and myocardial lipomatous metaplasia contribute further to arrhythmic susceptibility.^46,47^ It remains unclear whether these factors, or additional ischaemia, take precedence in triggering VA in remodelled hearts after MI. It is conceivable that HRP can be beneficial in preventing arrhythmias when triggered by ectopic activity in absence of significant ischaemia, but this remains to be elucidated.

### Limitations

A major limitation was that MI was induced in healthy pigs, presenting a very different phenotype from that of the post-MI ICD carrier. Besides the intricate remodelling that occurs after MI, the comorbidities commonly found in this patient population, such as obesity, hypertension, and diabetes, are currently lacking. This obviously hampers translation of the found effect of HRP on arrhythmic outcomes to the human ICD population with ischaemic cardiomyopathy. A different model closer to the clinical situation should be used for future studies on this objective.

Secondly, differences between porcine and human properties should be considered. Despite a close resemblance between porcine and human coronary anatomy, significant differences in the position of the heart in the chest, anatomy of the conduction system and electrophysiological properties exist.^48^ The resulting abnormalities of the ECG, such as a prolonged AV delay and unstable electrophysiology resulting in increased susceptibility for VA, should be considered for translation of the results.

Moreover, the use of general anaesthesia and amiodarone/dronedarone is known to influence arrhythmogenesis. Current study did not include a control group without amiodarone or dronedarone, as prophylaxis is necessary to prevent refractory VF in this model.^49,50^ Its effect on STV in the ischaemic pig model is not known. However, in the CAVB dog model, amiodarone prolonged the QTc-interval, while STV remained stable.^26^ At the worst, amiodarone and dronedarone suppressed the progression of STV towards VA, since an interaction between the occurrence of VA and STV is known. Nonetheless, an increase in STV prior to VT/VF was still demonstrated. Additionally, measurements within each pig were acquired under consistent circumstances, ensuring the reliability of serial comparisons and comparison between STV-ARI_auto_ and STV-QT_FSA_.

Furthermore, different regimens in pre-medication, pre-conditioning, and induction have been followed for males and females (Study Part 1) because of the difference in VF susceptibility.^51^ This might have influenced arrhythmic outcomes. However, the aim of Part 1 was to validate the previously described increase in STV-QT towards VF during acute MI,^20^ not to assess arrhythmic outcomes. As we have replicated the progression of STV-QT before VF and *Figure 3* shows no skewing between male and female subjects, the different regimens should not hamper the conclusions drawn from Part 1. More so, STV-QT’s ability to indicate imminent VF despite population heterogeneity highlights its robustness. For Part 2, only female pigs were included not to confound the arrhythmic outcomes.

Another significant limitation arises from the inability to determine STV during pacing due to interference of the pace spike in the T-wave. Consequently, only four measurements involving the T-wave were obtainable prior to VT/VF in Part 2 of the study. As a result, certain findings regarding STV progression towards VT/VF could not be replicated between Part 1 and Part 2 of the study and the effect of pacing on STV could not be assessed. Again, an alternative model closer to the clinical situation should be employed to address these issues.

As all pigs developed VA upon MI, no control subjects were included in present work to differentiate between an STV-increase related to arrhythmia or as consequence of ischaemia. However, the study referred to earlier did include STV-QT values of pigs that remained free of VF after MI.^20^ A difference in STV-QT between VF+ and VF− pigs at matched timepoints is demonstrated after occlusion, most pronounced 1 min before VF. As the data from the current study are in agreement with the data from Amoni *et al.*,^20^ it is plausible that the increase in STV is indeed related to VA.

Finally, the greater decrease in EF in the intervention group of Part 2 could be confounded by the larger number of shocks administered in this group. However, an aggravating effect of elevated heart rate on infarct size has been established^24^ and it is therefore fair to assume HRP as the main driver of the more pronounced decreased EF. An interaction between HRP, aggravating ischaemia, increasing the occurrence of VF during which circulation ceases, aggravating ischaemia, and so on is likely to reinforce the process.

### Future directions

As STV-ARI_auto_ measurements have shown promise in predicting impending VA in multiple pre-clinical models, further development should move towards clinical application. An important next step entails expansion of the algorithm to autonomically attenuate for confounding factors as noise, ectopy, and morphology changes, preferably on human signals. Integration into ICDs would be optimal, allowing simultaneous consideration of factors like circadian variations, daily activities, sympathetic stimulation, anti-arrhythmic drugs, and different ICD vectors. Furthermore, it would enable assessment of the events leading up to spontaneous VA. As such potential indicators of HRP feasibility can be identified: the presence of ST-elevation might suggest an argument against application of HRP, whereas triggered activity with R-on-T might indicate an argument in favour.

For further exploration of STV-guided HRP as a preventive measure against VA in ischaemic cardiomyopathy, an alternative experimental model should be employed. Specifically, a remodelled heart model where spontaneous VA occur and with a shorter AV delay. As such STV can be explored during pacing, which offers an opportunity to assess the efficacy of the pacing rate and adjust it in a closed-loop system with negative feedback. This would allow constant evaluation and optimization of the pacing rate.

### Conclusion and clinical implications

Automatically measured STV-ARI_auto_ values on the intracardiac EGM increase before the onset of VA alike the gold-standard STV-QT_FSA_ in a porcine model of MI, indicating imminent arrhythmias. This highlights the potential of arrhythmic risk monitoring by cardiac devices through STV-ARI_auto_ measurements and subsequent initiation of preventive strategies when necessary. As such ICD shocks could be avoided, which can positively impact patients’ quality of life. Continuous HRP during onset of acute MI did not improve arrhythmic outcomes, but might have anti-arrhythmic properties in remodelled hearts through suppression of triggering events. In ensuing validation studies, the accuracy of STV-ARI_auto_ to predict impending VA needs to be evaluated clinically and the feasibility of STV-guided HRP to prevent VA should be explored in a non-porcine model of ischaemic cardiomyopathy.

## Translational perspective

This study demonstrated that short-term variability (STV) of repolarization increases prior to ventricular arrhythmias during ischaemic circumstances in pigs. This indicates that monitoring of STV may be useful for arrhythmic risk monitoring in the acute setting after myocardial infarction. To integrate this into cardiac devices, a previously developed method for automatic real-time STV measurements on the intracardiac electrogram (STV-ARI_auto_) was evaluated. STV-ARI_auto_ values increase before the onset of VA alike the gold-standard. This highlights the potential of arrhythmic risk monitoring by cardiac devices through STV-ARI_auto_ measurements and subsequent initiation of preventive strategies when necessary. Consequently, shocks to terminate life-threatening ventricular arrhythmias by the implantable cardioverter defibrillator could be avoided, positively impacting patients’ quality of life. Continuous high rate pacing during onset of acute myocardial infarction did not improve arrhythmic outcomes. This suggests that future application of STV-guided high-rate pacing should be avoided during acute ischaemia, but its use in the remodelled heart should be further explored.

## Authors’ contributions

V.L., A.S., M.A.V., and M.M. came up with the concept and designed the study. Data collection and measurements were completed by V.L., A.S., and H.D.M.B. Data analysis was performed by V.L., A.A.H., A.S., B.H.R.v.d.L., and H.D.M.B. The results were interpreted by V.L., A.A.H., A.S., M.A.G.v.d.H., M.A.V., G.J.J.B., and M.M. Article drafting was performed by V.L., M.A.G.v.d.H., and M.M. All authors approved of the final version of the manuscript.

## Data Availability

The data underlying this article will be shared on reasonable request to the corresponding author.
